# Cortisol stress response after musculoskeletal surgery: a narrative review

**DOI:** 10.1530/EOR-2024-0126

**Published:** 2025-04-01

**Authors:** Maximilian M Menger, Tina Histing, Matthias W Laschke, Sabrina Ehnert, Tim Viergutz, Johann Fontana

**Affiliations:** ^1^ Department of Trauma and Reconstructive Surgery, BG Trauma Center Tuebingen, Eberhard Karls University Tuebingen, Tuebingen, Germany; ^2^ Institute for Clinical & Experimental Surgery, Saarland University, Homburg, Germany; ^3^ Department of Trauma and Reconstructive Surgery, BG Trauma Center Tuebingen, Siegfried Weller Institute for Trauma Research, Eberhard Karls University Tuebingen, Tuebingen, Germany; ^4^ Department of Anesthesiology and Intensive Care Medicine, BG Trauma Center Tübingen, Tübingen, Germany; ^5^ Medical Faculty of Mannheim, University of Heidelberg, Mannheim, Germany

**Keywords:** cortisol, stress response, surgery, inflammation, trauma

## Abstract

- Trauma induced by surgery stimulates a neuroendocrine stress response, substantially increasing cortisol levels in the post-surgical setting. This has substantial effects on metabolism, water and electrolyte balance as well as on the cardiovascular, nervous and immune systems.
- While there are valid data on cortisol level courses in a variety of specific pathologies, such as septic shock, acute respiratory distress syndrome, bacterial meningitis, cardiac arrest, community-acquired pneumonia and influenza, there is a persisting lack of data on the cortisol stress response after musculoskeletal surgery.
- The present review provides an overview of the current state of research regarding trauma-induced cortisol response after musculoskeletal interventions, including both elective orthopedic surgery and trauma surgery.
- Trauma induced by musculoskeletal surgery triggers a cortisol response, which varies significantly depending on the type of surgery and its invasiveness. Notably, elective orthopedic procedures demonstrate a smaller range of cortisol levels compared to musculoskeletal trauma and surgery.
- In the future, high-quality prospective trials need to analyze the factors that may modulate the adequate adrenal response to stress, such as preoperative long-term treatments with glucocorticoids, as well as the potential impact of low cortisol levels and perioperative cortisol substitution therapy on pain management, blood requirements, catecholamine dependency, delirium and mortality after musculoskeletal surgery.

## Introduction

**Figure 1:**
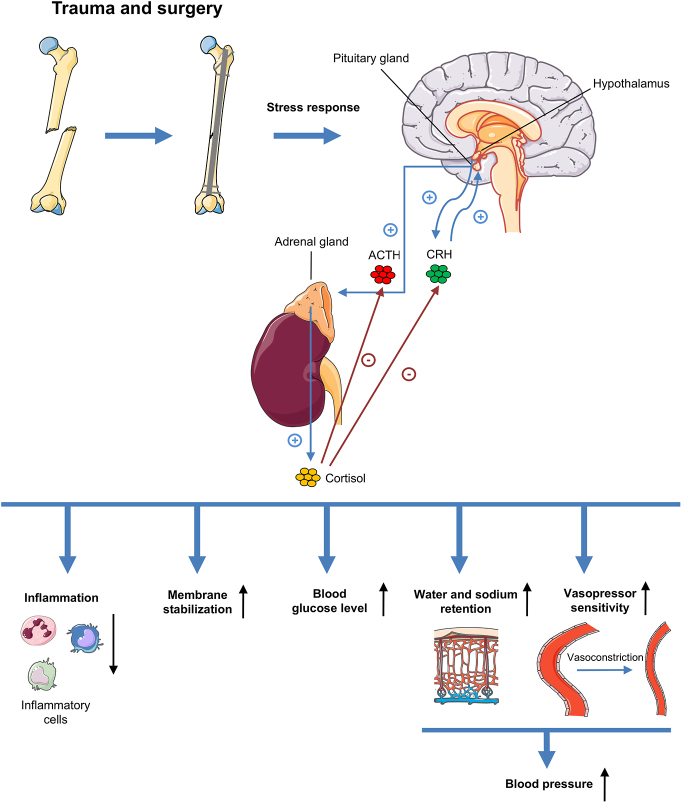
Trauma and subsequent surgery induce a stress response, resulting in the release of CRH from the hypothalamus. CRH promotes pituitary ACTH secretion, stimulating the release of cortisol from the adrenal cortex. Cortisol itself decreases CRH and ACTH secretion by a negative feedback loop. Moreover, it induces a variety of systemic effects, including an alleviation of inflammation with a reduction of inflammatory cells, membrane stabilization, elevation of blood glucose levels and an increase in blood pressure by stimulating water and sodium retention as well as enhancing vasopressor sensitivity with subsequent vasoconstriction.

Surgical interventions induce an upregulation of cortisol secretion by stimulating the hypothalamic–pituitary–adrenal (HPA) axis. Triggered by afferent nerves from the site of injury, the hypothalamus releases corticotropin-releasing hormone (CRH) and arginine vasopressin (AVP). This, in turn, promotes pituitary (ACTH) secretion, stimulating the release of cortisol from the adrenal cortex (Fig. 1) (1, 2).

Cortisol is a key regulator of the systemic inflammatory response after surgically induced trauma (3). This glucocorticoid exerts a plethora of effects, including anti-inflammatory actions, membrane stabilization, an increase in blood glucose levels and an elevation in blood pressure by sensitizing vascular smooth muscle cells to vasopressor agents and stimulating sodium and water retention (4, 5, 6, 7). Notably, the exact molecular mechanisms of cortisol action are highly complex, involving both genomic and non-genomic pathways (2).

Critical illness-related corticosteroid insufficiency (CIRCI) is a pathology characterized by a dysregulation of the systemic inflammatory response in a setting of critical illness, resulting from inadequate intracellular glucocorticoid-mediated anti-inflammatory activity with a corresponding negative impact on clinical outcomes. Accordingly, CIRCI guidelines recommend the supplementary use of glucocorticoids in specific pathologies and conditions, such as sepsis and toxic shock, acute respiratory distress syndrome, bacterial meningitis, cardiac arrest, community-acquired pneumonia and cardiac surgery (8, 9). In musculoskeletal surgery, however, there is a lack of knowledge regarding the expected perioperative cortisol levels and their significance for patient outcome and, thus, corresponding guidelines and treatment concepts are lacking. Moreover, an eventual pre-surgical glucocorticoid-induced HPA axis suppression, of any grade, would be able to directly impact the hormonal results. Accordingly, a deeper understanding of this surgery-induced cortisol stress response, together with early preoperative identification of those patients at higher risk of developing postsurgical relative adrenal insufficiency (10), such as those on long-term GC therapy before surgery, is of major clinical importance.

The present review provides an overview of the available literature on cortisol stress responses after musculoskeletal surgery, including standard values for perioperative cortisol levels and the potential influence of patient characteristics, such as age.

## Cortisol release after musculoskeletal surgery

### Elective orthopedic surgery

Due to considerably improved healthcare, the elderly population is steadily increasing worldwide (11, 12). The prevalence of osteoarthritis is up to 70% in women and 60% in men over the age of 65 years, making this condition a growing burden for orthopedic surgeons. Patients diagnosed with osteoarthritis suffer from substantial pain and severe functional deficits in the affected joint, resulting in a significant reduction in quality of life (13, 14). Total hip arthroplasty (THA) represents an effective method to reduce pain and improve mobility in these patients (15, 16).

Stratton and coworkers (17) investigated the role of plasma leptin in the early acute-phase response after surgical trauma. To do this, the authors also monitored cortisol levels in patients undergoing THA from the day before surgery until 8 days postoperatively. Their data show a significant increase in cortisol levels 24 h after surgery (686 ± 171 nmol/L; mean ± standard deviation (SD)) compared to preoperative values (479 ± 101 nmol/L) (Table 1). This was associated with increased mean leptin concentrations and significant hyperglycemia. Notably, between days 2 and 8 after surgery, cortisol levels showed no significant difference to preoperative baseline values (17). Høgevold *et al.* (18) analyzed platelet activation, catecholamine and cortisol response in patients undergoing uncemented THA. The authors found that cortisol levels started to rise 30 min after the beginning of the operation. Notably, cortisol values were still elevated at 48 h after surgery (242–869 nmol/L) compared to cortisol levels before anesthesia (242–390 nmol/L) (18) (Table 1). Of interest, the data also demonstrated a tendency for decreased noradrenaline levels from the end of surgery until 48 h postoperatively in patients receiving methylprednisolone when compared to the control group. These findings indicate an effect of perioperative steroid treatment on noradrenaline levels in patients undergoing orthopedic surgery (18). Notably, the number of patients included in the aforementioned studies is very low (*n* = 6–7). Therefore, ranges of cortisol levels vary substantially, especially in the report of Høgevold *et al.* (18), which makes a valid interpretation of the absolute numbers difficult.

**Table 1:** Studies analyzing the cortisol stress response after musculoskeletal surgery. The surgical intervention, number of patients, the cortisol level with the greatest dynamic to baseline values and the corresponding time point of investigation are illustrated.

Study/group	Surgical intervention	Patients, *n*	Cortisol level (nmol/L)^*^		Time point of investigation
			Value^*^	Change	
Stratton *et al.* (17)	THA	6	686 ± 171	↑	24 h po.
Høgevold *et al.* (18)	THA	7	242–869	↑	48 h po.
Van Boxsteal *et al.* (19)	THA				End of surgery
GA		25	529.7 ± 35.9	↑	
SA		25	237.2 ± 1.1	→	
Repo *et al.* (20)	Elective lumbar spine fusion	52		↓^‡^	24 h po.
Zhong *et al.* (22)	THA				6 h po.
Aged		31	701.9 ± 156.3	↑	
Middle-aged		30	807.1 ± 161.3	↑	
Juma *et al.* (31)	Multiple musculoskeletal interventions	16	698 ± 52^†^	↑	2 h po.
Kahveci *et al.* (32)	Lower limb surgery				24 h po.
GA		30	640.0 ± 350.3	↑	
EA		30	681.4 ± 292.4	↑	
Jain *et al.* (33)	Lower limb trauma				1 week po.
GA		178	1853.8 ± 253.8	↑	
EA		178	1233.1 ± 168.3	↑	
Nicholson *et al.* (34)	Pelvic reconstructive surgery	20	233 (179–534)	↓	48 h po.

THA, total hip arthroplasty; SEM, standard error of the mean; SD, standard deviation; GA, general anesthesia; SA, spinal anesthesia; EA, epidural anesthesia; po., postoperatively.

^*^ Values are presented as mean ± SD, as range or as mean (range).

^‡^ Significant decrease when compared to baseline values.

^†^ Value is mean ± SEM

In a recent study, Van Boxstael and coworkers (19) evaluated the potential benefits of spinal anesthesia in patients undergoing THA on the perioperative neuroendocrine stress response and perioperatively acquired muscle weakness when compared to general anesthesia. Their results show a significant increase in cortisol levels to 529.7 ± 35.9 nmol/L (mean ± SD) at the end of surgery when compared to preoperative values (342.1 ± 24.8 nmol/L) in patients receiving general anesthesia (Table 1). In addition, ACTH levels enhanced significantly at the end of surgery. Notably, cortisol and ACTH levels decreased again 24 h postoperatively. The increase in cortisol and ACTH was absent in patients receiving spinal anesthesia, indicating an attenuation of the neuroendocrine stress response in the immediate postoperative period (19).

Surprisingly, in a prospective clinical analysis, Repo *et al.* (20) found contradicting results regarding the cortisol response after orthopedic surgery in patients undergoing elective lumbar spine fusion. The authors’ results demonstrate a significant decrease in cortisol levels 24 h after surgery when compared to preoperative levels. In addition, ACTH levels dropped from 24 h until 72 h after surgery (20). These findings are in contrast with the observed neuroendocrine stress response in patients undergoing THA surgery. However, Repo *et al.* (20) did not monitor cortisol or ACTH levels during surgery and in the immediate postoperative period. Hence, an increase in cortisol levels in the first 24 h after lumbar spine fusion surgery cannot be excluded (20). Hence, further research is required to analyze the cortisol stress response after different orthopedic interventions during and immediately after surgery in order to gain valid reference values.

There is increasing evidence that age has a significant impact on the neuroendocrine stress response after surgery (21). Accordingly, Zhong *et al.* (22) investigated the cortisol and inflammatory stress response between aged and middle-aged patients undergoing THA. Aged (over 65 years) and middle-aged patients (40–65 years) demonstrated an increase in cortisol levels 6 h after surgery when compared to preoperative values (aged: 701.9 ± 156.3 nmol/L vs 314.8 ± 89.75 nmol/L (mean ± SD); middle-aged: 807.1 ± 161.3 nmol/L vs 291.9 ± 111.00) (Table 1). Interestingly, cortisol levels in aged patients were significantly lower 6 h after surgery when compared to the middle-aged population. Moreover, the data showed significantly higher levels of the pro-inflammatory cytokine interleukin (IL)-6 at 6 and 24 h after surgery and a significantly enhanced level of C-reactive protein in aged patients (22). This observation may be due to a relatively lower cortisol release in these patients, resulting in a higher inflammatory response. In fact, it has been reported that aged individuals exhibit higher levels of circulating pro-inflammatory cytokines, leading to a chronically increased inflammatory status, also referred to as ‘inflamm-aging’ (23, 24, 25). Furthermore, aging seems to be associated with impaired suprachiasmatic nucleus function, also affecting the hippocampus. Hence, its ability to modulate adrenocortical circadian rhythmicity (26) and stress response (27) is hampered, ultimately resulting in a decreased circadian fluctuation of cortisol secretion and a lower cortisol release after surgery (22, 28). Accordingly, the perioperative administration of corticoids may be particularly useful in aged patients undergoing musculoskeletal surgery. Previous studies have already demonstrated that perioperative administration of prednisolone and hydrocortisone significantly decreases IL-6 levels at 6 and 24 h after surgery (22, 29). Furthermore, perioperative administration of dexamethasone reduced postoperative nausea, pain and hospitalization length after THA (30).

In summary, the available scientific reports do not provide valid data to define reference values for the anticipated cortisol course after elective orthopedic surgery. Furthermore, no conclusion can be drawn concerning the question of whether an increased cortisol output after elective orthopedic surgery should be considered positive or negative. Therefore, future research is urgently needed to broaden knowledge on the exact pathophysiological mechanisms and consequences of a potentially ‘relative cortisol insufficiency’ in patients undergoing elective orthopedic surgery.

### Musculoskeletal trauma and surgery

There are only a few studies investigating the cortisol response after musculoskeletal trauma and surgery. Juma *et al.* (31) analyzed the effects of trauma and musculoskeletal surgery on cortisol and catecholamine levels between 2 and 168 h postoperatively in 16 patients with a wide range of diagnoses, including supracondylar femoral fractures, amputations as well as fractures of the radius and ulna. The data showed a significant increase in cortisol levels throughout the entire postoperative observation period. Two hours after surgery, the cortisol levels reached maximum values of 698 ± 52 nmol/L (mean ± SEM) when compared to preoperative levels (313 ± 54 nmol/L) (Table 1). This was associated with higher concentrations of adrenaline and noradrenaline (31).

The cortisol stress response of a more homogeneous study population was analyzed by Kahveci *et al.* (32). The authors assessed the stress hormone response in patients undergoing lower extremity surgery with epidural and general anesthesia. Their data revealed an increase in cortisol levels (681.4 ± 292.4 nmol/L (mean ± SD) (epidural anesthesia) and 640.0 ± 350.3 nmol/L (general anesthesia)) 24 h after surgery when compared to preoperative values (336.6 ± 143.4 nmol/L (epidural anesthesia); 347.6 ± 168.3 nmol/L (general anesthesia)) (32) (Table 1). By evaluating the effectiveness of on-arrival lumbar epidural analgesia for lower limb trauma pain management, Jain and coworkers (33) found a similar dynamic for cortisol levels after lower extremity trauma. Patients receiving epidural analgesia exhibited cortisol levels of 731.0 ± 168.3 nmol/L (mean ± SD), and patients receiving standard analgesia had levels of 780.7 ± 201.4 nmol/L, with a substantial increase when compared to baseline levels 24 h after surgery. Interestingly, cortisol levels were steadily increasing for one week after the intervention with significantly higher levels in patients with standard analgesia (1233.1 ± 168.3 nmol/L vs. 1853.8 ± 253.8 nmol/L). This indicates an attenuated stress response in patients with epidural analgesia (33) (Table 1).

However, there are also contradictory findings regarding cortisol release after trauma surgery. Nicholson *et al.* (34) investigated the hormonal and inflammatory response after major pelvic reconstructive interventions. Of note, the authors found no significant increase in cortisol levels throughout the entire postoperative observation period of 72 h. In fact, cortisol levels even showed a tendency of decreasing with the lowest concentration of 233 nmol/L (mean) 48 h after surgery when compared to preoperative levels (482 nmol/L) (Table 1). However, concentrations of the pro-inflammatory cytokines IL-6 and IL-10 rose significantly for 48 and 24 h, respectively, and returned to baseline values thereafter. Moreover, epinephrine values enhanced significantly 6 h postoperatively. One possible explanation for the missing cortisol stress response mentioned by the authors is the high doses of morphine that were needed for sufficient analgesia, a phenomenon that has already been reported in previous experimental and clinical studies (34, 35, 36, 37). In addition, it should be considered that acetabular and pelvic reconstructive surgery represents major trauma for the patient due to the necessity of extensive surgical approaches, such as the anterior ilioinguinal approach or the posterior Kocher-Langenbeck approach (38, 39, 40). Therefore, the observed unaltered cortisol levels in the study of Nicholson *et al.* (34) may represent insufficient cortisol release, resulting in ‘relative cortisol insufficiency’ in response to largely invasive surgery.

In summary, similar to elective orthopedic surgery, the available scientific reports describing cortisol release after musculoskeletal trauma and surgery do not provide enough valid data to define reliable reference values for the post-surgical course. Furthermore, one major possible confounder was ignored by all the above-mentioned studies, i.e., the impact of musculoskeletal trauma on the circadian rhythm of cortisol secretion. While there are data available demonstrating a circadian disruption in a variety of critically ill patients (41), there are only insufficient data available to determine if this phenomenon can also be transferred to patients after elective orthopedic or musculoskeletal trauma surgery (42). Another potential confounder, which was not sufficiently discussed by the above-mentioned studies, is the suitability of the pre-surgical cortisol level as a reference value for further evaluations of the cortisol course, considering the CIRCI described previously in different settings.

**Figure 2:**
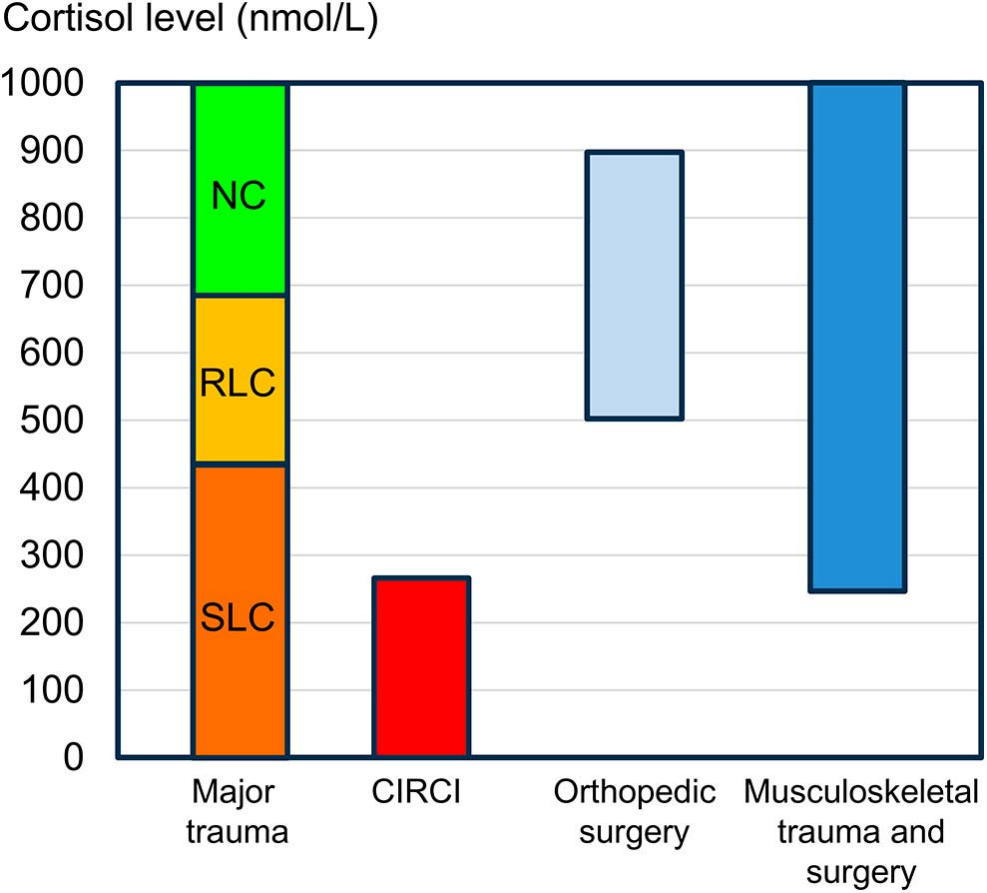
The cortisol response after major trauma can be divided into SLC: ≤413.8 nmol/L, RLC: 413.9–689.7 nmol/L and NC: >689.7 nmol/L. CIRCI is diagnosed by cortisol levels below 275.9 nmol/L. Cortisol levels with the highest dynamics after orthopedic surgery range approximately from 500 to 900 nmol/L and after musculoskeletal trauma and surgery from 250 up to over 1000 nmol/L.

Kwok *et al.* (7) investigated the impact of cortisol levels in acute critically ill trauma patients. The authors categorized ‘severe low cortisol’ (SLC) for patients with an initial total serum cortisol level less than or equal to 413.8 nmol/L, ‘relative low cortisol’ (RLC) for cortisol levels ranging from 413.9 to 689.7 nmol/L or ‘normal cortisol’ (NC) for cortisol levels higher than 689.7 nmol/L (7, 43). Their results demonstrate that patients with SLC were associated with larger blood product requirements within 24 h, increased vasopressor use within 72 h and an overall higher mortality. The cortisol levels after musculoskeletal surgery ranged in most cases, according to the above-mentioned definition, from relative RLC to NC levels. One exception represents the results of Nicholsan *et al.* (34), who found cortisol levels less than 413.8 nmol/L 48 h postoperatively in patients undergoing major reconstructive pelvic surgery with a mean ‘injury to surgery’ interval of 11 days. These findings may indicate an insufficient cortisol response in these patients. In fact, the Society of Critical Care Medicine (SCCM) and the European Society of Intensive Care Medicine (ESICM) suggest the diagnosis of CIRCI by a random total cortisol level below 275.9 nmol/L (8, 44) (Fig. 2). Accordingly, future studies need to evaluate the potential benefits of perioperative corticoid treatment in patients exhibiting SLC levels after or before musculoskeletal surgery.

## Conclusion and perspectives

Trauma induced by musculoskeletal surgery induces a cortisol response, which varies significantly between the type of surgery and invasiveness. Elective orthopedic surgery, such as THA, induces cortisol levels ranging from approximately 500 nmol/L up to 900 nmol/L. This cortisol stress response attenuates from the end of surgery until 48 h after surgery (Table 1). Musculoskeletal trauma and subsequent surgery results in a higher range of postoperative cortisol, ranging from SLC levels up to values above 1000 nmol/L. Of note, there is evidence that, in these cases, the cortisol stress response can persist for up to one week. Corresponding to the findings of Kwok *et al.* (7) and the CIRCI guidelines (8, 9), a cortisol value below 413.8 nmol/L on admission or a cortisol value below 275.9 nmol/L after trauma or elective musculoskeletal surgery might indicate a relevant cortisol deficit (Fig. 2). In this case, perioperative cortisol substitution should be taken into consideration. However, high-quality prospective trials analyzing the potential impact of low cortisol levels and perioperative cortisol substitution therapy on pain management, blood requirement, catecholamine dependency, delirium and mortality after musculoskeletal surgery are still lacking. Moreover, the impact of patient characteristics on cortisol levels during the perioperative setting, such as age, sex, preoperative long-term treatment with glucocorticoids, and comorbidities, is largely unknown. Only with this additional knowledge will it be possible to identify patients at a high risk of developing peri- and post-surgical relative adrenal insufficiency and establish reliable guidelines to improve patient outcomes after musculoskeletal trauma.

## Declaration of interest

The authors declare that there is no conflict of interest that could be perceived as prejudicing the impartiality of the work.

## Funding

We acknowledge support from the Open Access Publication Fund of the University of Tuebingen.
